# Trans-fistulous pancreatoscopy-guided pancreatic duct stone extraction after endoscopic ultrasound-guided pancreaticogastrostomy in post-Whipple patient

**DOI:** 10.1055/a-2780-8700

**Published:** 2026-02-05

**Authors:** MengHan Wang, YiJun Chen, WenHai Wang

**Affiliations:** 1 Department of Gastroenterology, Beijing Friendship Hospital, Capital Medical University, Beijing, China

Pancreatic ductal adenocarcinoma is highly lethal, and pancreaticoduodenectomy leaves a pancreaticojejunal anastomosis that may later stricture, causing obstructive pancreatitis and pancreatic duct stones that are difficult to treat endoscopically in altered anatomy. Endoscopic retrograde cholangiopancreatography (ERCP) often fails to access the pancreatic remnant in this setting, and EUS-guided pancreatic duct drainage has emerged as a minimally invasive alternative 1 .

We present a 60-year-old man 1 year after Whipple resection for pancreatic head cancer who developed recurrent postprandial abdominal pain. Cross-sectional imaging and magnetic resonance cholangiopancreatography revealed a dilated main pancreatic duct with a stone at the pancreaticojejunal anastomosis. Two ERCP attempts, including enteroscopy-assisted ERCP, failed to identify the pancreaticojejunal opening.

Using a echoendoscope from the stomach, we created an EUS-guided pancreaticogastrostomy with a double-pigtail stent. Then, after tract maturation, we performed antegrade pancreatoscopy through the fistula to extract the pancreatic duct stone, dilate the anastomotic stricture, and place a trans-anastomotic stent ( Video 1 ). Symptoms resolved without complications, illustrating that EUS-guided pancreaticogastrostomy with antegrade therapy can obviate reoperation in post-Whipple patients with pancreaticojejunal anastomotic strictures and pancreatic duct stones.

The video shows endoscopic ultrasound–guided pancreaticogastrostomy creation, followed by trans-fistulous pancreatoscopy through the mature tract, direct visualization of pancreatic duct stones, and pancreatoscopy-guided stone extraction.Video 1

Endoscopy_UCTN_Code_TTT_1AS_2AD
